# Curcumin-Induced Molecular Mechanisms in U-87 MG Glioblastoma Cells: Insights from Global Gene Expression Profiling

**DOI:** 10.3390/molecules30102108

**Published:** 2025-05-09

**Authors:** Nicole Tendayi Mashozhera, Chinreddy Subramanyam Reddy, Yevin Nenuka Ranasinghe, Purushothaman Natarajan, Umesh K. Reddy, Gerald Hankins

**Affiliations:** 1 Department of Biology, West Virginia State University, Institute, WV 25112, USA; nmashozhera@wvstateu.edu (N.T.M.); subramanyam.chinreddy@wvstateu.edu (C.S.R.); yevin.n.ranasinghe@gmail.com (Y.N.R.); pnatarajan@wvstateu.edu (P.N.); 2 Department of Agriculture, Food, and Resource Sciences, University of Maryland Eastern Shore, Princess Anne, MD 21853, USA

**Keywords:** glioblastoma-U87 cells, natural compound, curcumin, apoptosis, RNA-seq

## Abstract

Curcumin, a major phytochemical derived from Curcuma longa, has been shown to enhance the efficacy of chemotherapeutic agents such as doxorubicin, 5-fluorouracil, and cisplatin by overcoming drug resistance, making it a promising adjunct in the treatment of glioblastoma. However, the global gene-expression changes triggered by curcumin in glioblastoma remain underexplored. In this study, we investigated the effects of curcumin on human glioblastoma (U87 MG) cells, where it significantly reduced cell viability and proliferation in a dose- and time-dependent manner and induced apoptosis without affecting senescence. Transcriptomic analysis revealed 5036 differentially expressed genes, with pathway enrichment identifying 13 dysregulated cancer-associated pathways. Notably, curcumin modulated several key regulators involved in MAPK, Ras, TGF-β, Wnt, Cytokine, and TNF signaling pathways. Several apoptosis and cell cycle-associated genes, including PRKCG, GDF7, GDF9, GDF15, GDF5, FZD1, FZD2, FZD8, AIFM3, TP53AIP1, CRD14, NIBAN3, BOK, BCL2L10, BCL2L14, BNIPL, FASLG, GZMM, TNFSF10, TNFSF11, and TNFSF4, were significantly altered. Several pro-apoptotic and anti-BCL, cell-cycle-regulated genes were modulated following curcumin treatment, emphasizing its potential role in curcumin-mediated anti-tumor effects. This study provides insight into the molecular mechanisms underlying curcumin’s action against glioblastoma.

## 1. Introduction

*Glioblastoma multiforme* is the most common primary malignancy of the brain, with an incidence of 3.2 in 100,000 [1]. Glioblastoma remains a formidable challenge, with a five-year survival rate below 10% despite aggressive treatment [2]. The hallmarks of glioblastoma include aggressive and diffuse proliferation, and resistance to apoptosis-inducing drugs [3]. Tumor drug resistance is a major cause of treatment failure, driven by cellular heterogeneity, diverse molecular signatures, and variable drug responsiveness [4]. Conventional chemotherapy fails to achieve complete remission and induces toxicity, harming normal cells and causing severe side effects [5].

Plant-derived compounds, known for their pleiotropic anti-cancer effects, have gained interest in overcoming multi-drug resistance [6,7]. Natural products like resveratrol, quercetin, EGCG, and curcumin enhance chemotherapy by sensitizing tumor cells through antioxidant, anti-inflammatory, immune-modulatory, and apoptosis-inducing mechanisms, boosting efficacy without added toxicity [8].

Curcumin, a yellow pigment from Curcuma longa, is a well-studied anti-cancer agent with low toxicity and therapeutic potential [9]. Preclinical studies show that curcumin enhances the efficacy of chemotherapeutic drugs like sulfinosine, 5-FU, doxorubicin, oxaliplatin, and cisplatin, often sensitizing drug-resistant cells [10]. In prostate cancer, curcumin with docetaxel improved anti-proliferative and apoptotic effects [11]. It also boosted 5-FU efficacy in colon, breast, and gastric cancers [12,13]. In a pancreatic cancer xenograft, curcumin with gemcitabine significantly reduced tumor volume by enhancing anti-proliferative and anti-angiogenic pathways [14]. Clinical trials suggest daily curcumin supplementation exerts anti-inflammatory and immune-modulatory effects in various cancers [15].

Curcumin modulates key pathways in tumor initiation, promotion, and progression. It suppresses carcinogenesis by inducing apoptosis, arresting the cell cycle, and inhibiting metastasis, invasion, and angiogenesis [16]. In glioblastoma, curcumin suppresses growth and chemoresistance via AP-1 and NFκB transcription factors, which regulate cell proliferation, apoptosis, and inflammation. In lung cancer, it inhibits metastasis by targeting matrix metalloproteinases (MMP-2 and MMP-9) and vascular endothelial growth factor (VEGF), crucial factors in tumor invasion. For hepatocellular carcinoma, curcumin’s suppression of proliferation and induction of apoptosis occurs through modulation of the Wnt signaling pathway. Similarly, in non-small-cell lung cancer, it impedes migration and invasion through the up-regulation of miR-206 and suppression of the PI3K/AKT/mTOR pathway. Across these studies, curcumin’s multifaceted role in regulating critical molecular signaling pathways and ncRNAs make it a potential multi-targeted therapeutic agent that can be leveraged in cancer prevention and treatment, enhancing efficacy and overcoming resistance mechanisms [17,18,19,20,21].

Although extensive research has revealed curcumin’s anti-carcinogenic properties and its potential to support cancer therapy, studies examining its impact on global gene expression via transcriptome profiling in various cancer cells remain limited. High-throughput RNA sequencing (RNA-seq) of curcumin-treated cells can elucidate the anti-proliferative and cell-death pathways associated with curcumin [22]. Recent studies have employed RNA-seq to profile the most significantly up- and down-regulated genes following curcumin treatment, followed by protein–protein interaction analysis and in silico molecular docking to identify novel curcumin targets [23]. Compared with targeted proteomic methods, integrating global pathway enrichment analyses with systemic studies yields a more comprehensive insight into the regulatory and functional dynamics driving curcumin-induced cytotoxicity in cancer, thereby enhancing its therapeutic application [24]. In two breast cancer cell lines, transcriptome profiling showed that curcumin primarily induced cell death via ferroptosis rather than apoptosis, suggesting that ferroptosis may be a more promising therapeutic target [25]. In contrast, in adrenocortical carcinoma cells and their xenograft model, curcumin induced apoptosis mainly through ER stress pathways with accompanying up-regulation of p38 and JNK/MAPK signaling [24].

Given the limited number of transcriptome profiling studies on curcumin, further research could enhance our understanding of its cytotoxic mechanisms in cancer [25]. Global gene expression analyses can identify novel genetic targets of curcumin, guiding future research efforts [26]. Accordingly, this study evaluated the impact of curcumin on global gene expression in the U87-MG human glioblastoma cell line using RNA-seq, with its cytotoxic effects confirmed via CCK-8 proliferation, apoptosis, and senescence assays.

## 2. Results

### 2.1. Curcumin Treatment Decreases U87 MG Cell Viability and Proliferation

**Figure 1:**
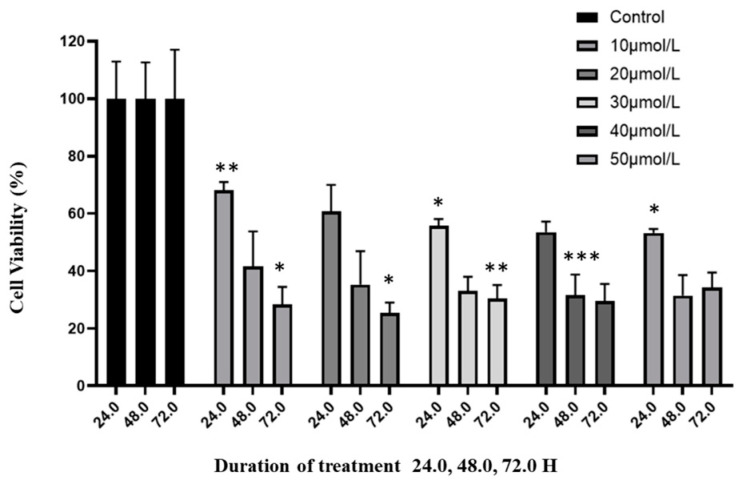
Effect of curcumin on glioblastoma cells. The anti-proliferative effects of increasing concentrations of curcumin on viability of U87 MG cells was assessed over a three-day period using the CCK-8 assay. Vehicle-treated cells were used as the negative control at each interval (100% viability). The viability is presented relative to the negative control. Values are means ± SD; *n* = 3; * *p* < 0.05 and ** *p* < 0.01, *** *p* < 0.001 (as compared with control).

The growth-inhibitory effects of curcumin on human glioblastoma cells were evaluated. Cell viability was assessed using the CCK-8 assay. Results demonstrated that curcumin reduced the viability and proliferation of U87 MG cells in a dose- and time-dependent manner (Figure 1). The median IC50 for curcumin was determined to be 20 μmol/L (Supplementary Figure S1). Based on this, subsequent experiments were conducted using a treatment concentration of 20 μmol/L.

### 2.2. Curcumin Reduced Migration of U87 MG Cells

The effects of curcumin treatment on the migratory ability of U87 MG cells were assessed using the scratch wound healing assay. The potential for cells to migrate into the wound in vitro represents their metastatic ability in vivo. A scratch wound was made in the monolayer of U87 MG cells using a 20 µL pipette tip, and cells were treated with curcumin or vehicle. Quantitatively, the inhibition of migration was assessed by measuring gap closure after 24 h of treatment with curcumin or vehicle.

**Figure 2:**
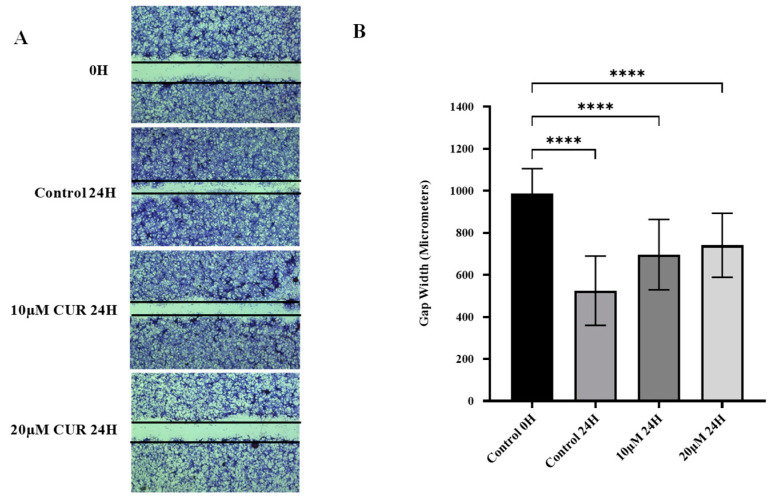
Effect of curcumin on migration of U87 MG cells. (**A**) The cell monolayer was scratched with a 20 µL pipette tip, photographed at 0 h, and treated for 24 h. (**B**) Gap closure was quantified by comparing gap width at 0 h and 24 h post-treatment with vehicle or curcumin. Data are mean gap width of six replicates ± SD. One-way ANOVA was used to examine statistical differences followed by Tukey’s honestly significant difference test for post hoc testing for differences between treatment groups. **** Significant at *p* < 0.0001.

Treatment of the cells with 10 and 20 μmol/L curcumin significantly reduced the number of cells that migrated into the wound after 24 h compared with vehicle-treated cells (*p*-value < 0.0001) (Figure 2). Furthermore, the rate of gap closure was reduced when the curcumin concentration was increased from 10 to 20 μmol/L (*p*-value < 0.01). The results of this study demonstrate that curcumin exhibits anti-migratory effects in U87 MG cells, and this effect is dose-dependent.

### 2.3. Curcumin Induced Apoptosis but Not Senescence in U87 MG Cells

**Figure 3:**
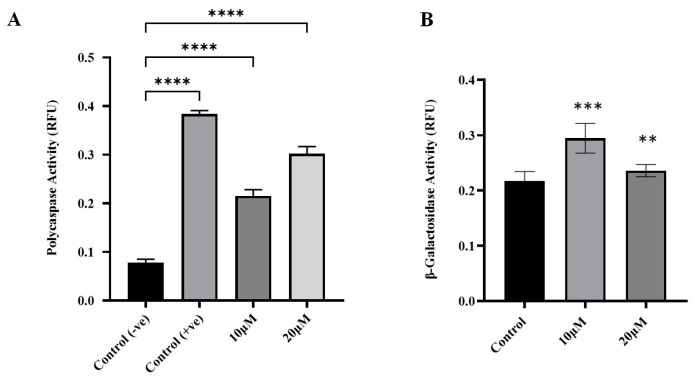
Effects of curcumin on apoptosis and senescence in U87MG cells. (**A**) Apoptosis induction was determined by poly-caspase activation following curcumin treatment for 24 h. (**B**) Curcumin-induced senescence was measured using the SA-β-galactosidase assay. Data are means ± SD of three independent replicates. One-way ANOVA tests were performed to determine the statistical significance of differences between control and treatment means. Tukey’s honestly significant difference tests were used for post hoc analysis of multiple comparisons. **** Significant at *p* < 0.0001. *** Significant at *p* < 0.001. ** Significant at *p* < 0.01.

To investigate the effects of curcumin on apoptosis and senescence in human glioblastoma cells, curcumin was administered to cells, and these cells were incubated for 24 h. Following incubation, apoptotic cells were identified through poly-caspase activity using the FAM-FLICA poly-caspase assay. When compared with untreated cells, curcumin-treated cells had significantly higher poly-caspase activity (*p*-value < 0.0001) (Figure 3A). Increasing curcumin concentration from 10 to 20 μmol/L also increased the rate of poly-caspase activity, indicating that curcumin-induced apoptosis in U87 MG cells was dose-dependent.

Senescent cells are identified through elevated lysosomal activity, which can be biochemically assayed through the activity of β-galactosidase enzyme [27]. In this study, curcumin-induced senescence was assayed using the SA-β-galactosidase assay kit. Curcumin treatment did not significantly induce senescence in U87 MG cells (*p* > 0.05 when compared to control) (Figure 3B).

### 2.4. Curcumin Induced Differential Gene Expression in U87 MG Cells

**Figure 4:**
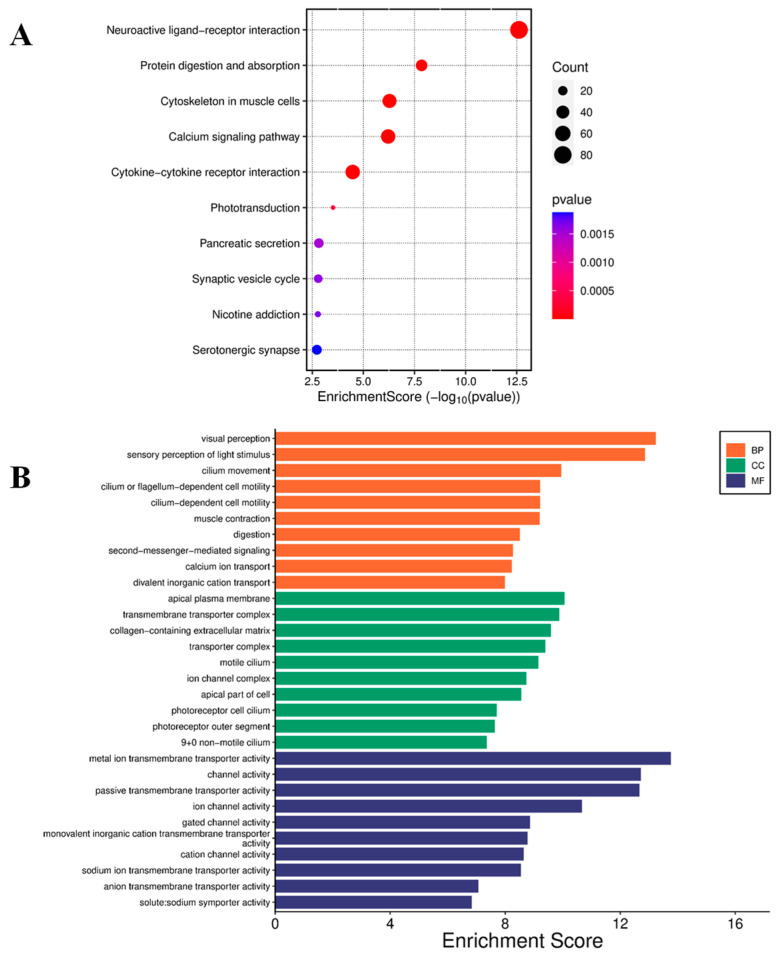
Enrichment analysis of up-regulated genes. (**A**) Bubble plot for KEGG pathway enrichment of the top 50 up-regulated genes. Each circle represents an enriched function. The gene enrichment ratio is assigned to the *x*-axis, and the descriptions of the pathways are assigned to the *y*-axis. The area of the circles is proportional to the number of genes assigned to the term, and the color to the adjusted *p*-value. (**B**) GO of three ontologies enriched in curcumin-induced up-regulated genes. The width of each bar represents enrichment score of genes (−log_10_(*p*-value)). BP = biological process, CC = cellular component, MF = molecular function.

The impact of curcumin on global gene expression in U87 MG cells was analyzed using RNA sequencing (RNA-seq), including principal component analysis (Supplementary Figure S2). Treatment with 20 μmol/L curcumin resulted in the differential expression of 5036 genes, with 3418 genes up-regulated and 1618 down-regulated. Kyoto Encyclopedia of Genes and Genomes (KEGG) pathway analysis of the top 50 up-regulated genes identified enrichment in pathways related to extracellular matrix (ECM)–receptor interaction, focal adhesion, cell adhesion molecules, Notch signaling, NF-κB signaling, Jak-STAT signaling, and choline metabolism in cancer (Figure 4A). Gene Ontology (GO) analysis revealed significant enrichment in molecular functions, including metal ion transmembrane transport, ion channel activity, channel activity, and gated channel activity (Figure 4B).

**Figure 5:**
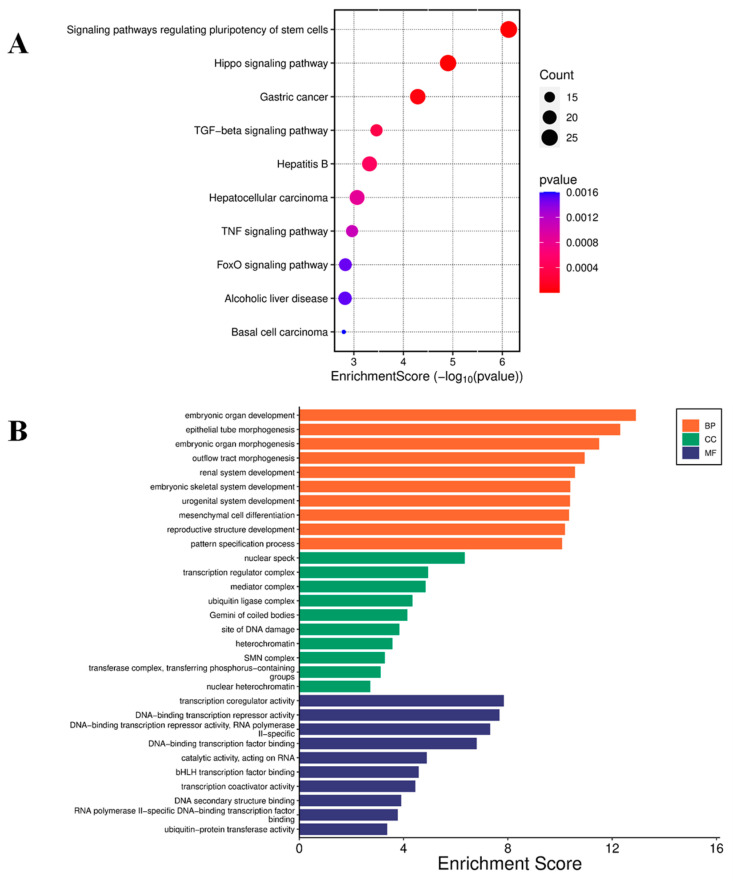
Enrichment analysis of down-regulated genes. (**A**) Bubble plot for KEGG pathway enrichment of the top down-regulated genes. Each circle represents an enriched function. The gene enrichment ratio is assigned to the *x*-axis and the description of the pathways to the *y*-axis. The area of the circles is proportional to the number of genes assigned to the term, and the color to the adjusted *p*-value. (**B**) GO of three ontologies enriched in curcumin-induced down-regulated genes. The width of each bar represents enrichment score of genes (−log_10_(*p*-value)). BP = biological process, CC = cellular component, MF = molecular function.

Conversely, KEGG pathway analysis of the top 50 down-regulated genes revealed involvement in pathways including O-glycan biosynthesis; Notch signaling; glycine, serine, and threonine metabolism; glycolysis and gluconeogenesis; and cytokine–cytokine receptor interaction (Figure 5A). GO analysis further highlighted enrichment in molecular functions, including DNA-binding transcription repressor activity, DNA-binding transcription factor binding, transcription coactivator activity, and catalytic activity acting on RNA (Figure 5B).

Further analysis of the 50 most significantly altered KEGG pathways identified 13 pathways strongly linked to tumorigenesis, cancer progression, and immune evasion. These included cytokine–cytokine receptor interaction; pathways in cancer; MAPK signaling; regulation of stem cell pluripotency; TGF-β signaling; breast cancer; Ras signaling; IL-17 signaling; PD-L1 expression and PD-1 checkpoint in cancer; proteoglycans in cancer; Wnt signaling; gastric cancer; and viral protein interaction with cytokines and cytokine receptors. Among these, several tumor-suppressive pathways stood out. Genes within MAPK, Ras, TGF-β, and Wnt pathways, and cytokine–cytokine receptor signaling, such as PRKCG (+8.81), GDF7 (+7.78), GDF5 (−2.68), FZD1 (+2.62), FZD2 (+2.95), and FZD8 (−3.18) are hypothesized to have contributed to curcumin’s anti-tumorigenic effects in this study. Additionally, the well-known tumor suppressor gene RUNX3 was significantly up-regulated (+8.93) in curcumin-treated cells, further supporting its potential role in curcumin-mediated tumor suppression.

## 3. Discussion

Curcumin, a polyphenol from Curcuma longa, is a well-studied natural compound that modulates tumor initiation, progression, and promotion in various cancers [28]. In glioblastoma, curcumin suppresses oncogenic signaling, induces multimodal cell death, and modulates immune components within the tumor microenvironment (TME) [29]. This study examines the impact of curcumin on global gene expression and its effects on proliferation, migration, apoptosis, and senescence in U87MG glioblastoma cells.

### 3.1. Curcumin Suppresses Viability and Migration of U87 MG Cells

Curcumin inhibited U87 MG cell proliferation in a dose- and time-independent manner, aligning with previous studies. RNA sequencing suggested that curcumin suppresses malignancy by inhibiting oncogenic pathways, including the Wnt/β-catenin, PI3K/Akt/mTOR, NF-κB, and TGF-β pathways [30,31]. Also, in our study, crucial pathways for cell survival—the PI3K-Akt (CDK6 (−1.55) CSF3 (−1.55) LPAR1 (−1.46) EFNA5 (−1.13) PHLPP2(−1.17) PHLPP1 (−1.11) GHR (−1.12) IRS1 (−1.71) KDR (−1.16) KRAS (−1.65) MCL1 (−1.80) PDGFA (−1.59) PDGFRA (−1.66) PIK3R1 (−1.88) PRKAA1 (−1.09) BDNF (−1.85) SGK1 (−1.44 fold) SOS2 (−1.05) TLR2 (−1.03) TLR4 (−2.69) CCNE1 (−1.27) CCNE2 (−1.73)), Wnt (FRAT2 (−1.12) FZD2 (−2.95) APC (−1.06) SMAD3 (−1.67) WNT5A (−1.82) WNT7B (−1.07) FZD5 (−1.99) FZD1 (−2.62) FZD7 (−1.93) FZD8 (−3.18) ROCK2 (−1.17) mTOR, (FZD2 IRS1 KRAS PIK3R1 PRKAA1 SGK1 SKP2 (−2.19) SOS2 WNT5A WNT7B FZD5 FZD1 FZD7 FZD8)), TNF signaling (TAB1 (−1.13) CEBPB (−1.5) MAPK14 (−1.49) JAG1 (−1.04) IL1B (−1.07) LIF (−1.49) PIK3R1 BCL3 (−1.14) TRAF5 (−1.59) RIPK1 (−1.68) FADD (−2.88) BAG4 (−1.04)), and TGF-β signaling (RGMB (−1.99) ID1 (−1.86) ID2 SMAD3 (−1.67) SMAD5 (−1.03) PITX2 (−1.7) SMURF2 (−1.04) BMP2 (−1.62) SP1 (−1.7) TGFBR1 (−1.02) TGFBR2 (−1.98) GDF5 (−2.68) ACVR1B (−1.48) ACVR2A (−1.2) NOG(−3.61)) pathways—were down-regulated, which is essential for cancer cell survival, metastasis, and drug resistance. Additionally, curcumin induces G2/M cell cycle arrest by modulating cell cycle regulators, thereby curbing tumor growth both in vitro and in vivo [32]. The current study also demonstrated cell-cycle-arrest-related cyclin-dependent kinase (CDK6 (−1.56), CCNE1 (−1.27), CCNE2 (−1.73), KRAS (−1.65), PIK3R1 (−1.88) PDGFA (−1.59), KDR (−1.16)) anti-angiogenic effects and TLR4 (−2.4) and IRS1 (−1.71) inflammation reduction effects. Targeting these pathways effectively induces apoptosis and prevents cancer progression.

Scratch wound assays revealed a significant (*p* < 0.0001) reduction in U87 MG cell migration following curcumin treatment, with greater inhibition observed at 20 μmol/L (*p* < 0.05). Curcumin’s anti-migratory effects involve the PI3K/Akt, NF-κB, ERK, and JAK-STAT signaling pathways, which down-regulate MMPs, VEGF, and ICAM-1 [18,32,33,34]. In glioblastoma, curcumin inhibited STAT3 phosphorylation, reducing MMP-9, Snail, and Twist expression [35], and suppressed MMPs via MAPK inhibition [36]. A study by Abdullah Thani et al. reported that decreased levels of MMP-2, -9, -14, -15, -16, -17, -24, and -25, further hindering glioblastoma migration [37]. Given glioblastoma’s aggressive migration at diagnosis, these findings underscore the therapeutic potential of curcumin.

In our study, JAK STAT (CSF3, GHR, LIF, MCL1, PDGFA, PDGFRA, PIK3R1, SOS2) and Nf-kB (TAB1, IL1B, TLR4, TRAF5, TRAF6, RIPK1)-related genes were also down-regulated.

### 3.2. Curcumin Triggers Apoptosis in U87 MG Glioblastoma Cells

**Figure 6:**
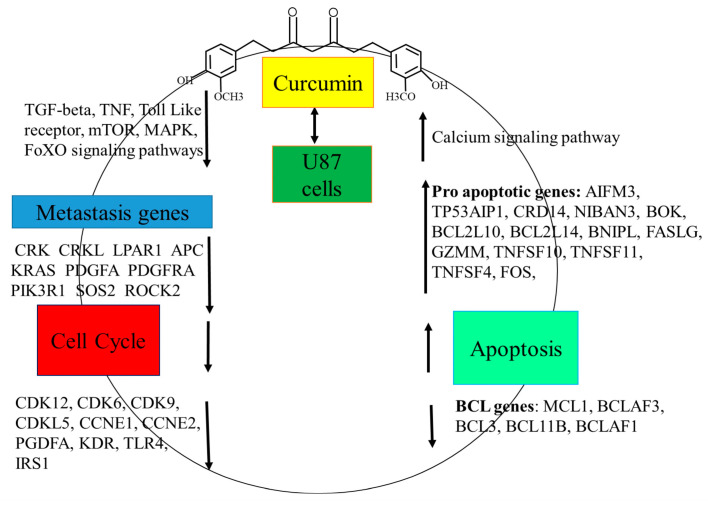
Thematic illustration. Curcumin treatment in U87 glioblastoma cells modulates global gene expression, leading to cell cycle arrest and apoptosis through the coordinated up-regulation and down-regulation of multiple signaling pathways and their associated genes.

Resistance to apoptosis is a major challenge in glioblastoma treatment, making apoptosis-inducing compounds crucial [31]. Curcumin-induced apoptosis was assessed via poly-caspase activity using the FAM-FLICA probe, which irreversibly binds active caspase-3, -6, -7, -8, -9, and -10. After 24 h, curcumin significantly increased poly-caspase activity, with higher activation at 20 μmol/L compared with 10 μmol/L, consistent with previous findings [38]. Curcumin promotes apoptosis by modulating apoptosis-related proteins, activating intrinsic (mitochondrial) (AIFM3 (5.3), BOK (2.5), BCL2L10, and (BCL2L14) and extrinsic (death-receptor) (FASLG (4.6), TNFSF10 (2.9), and TNFSF4 (1.7)) pathways, and mediating ER stress (TP53AIP1 (4.2), CRD14, NIBAN3 (3.8), BCL2L14, BNIPL, FASLG (4.6), GZMM, and TNFSF11 (6.4)) [31,39,40,41,42], Figure 6. It also enhances cleaved caspase-3, -8, and -9 expression in glioblastoma cells [38,43,44].

The effect of curcumin on senescence in U87 MG cells was examined using β-galactosidase activity. While glioma senescence can both suppress and promote malignancy in vivo [27], curcumin (10–20 μmol/L) did not induce senescence (*p* > 0.05). Prior studies suggest curcumin’s effects depend on concentration—low doses (≤10 μmol/L) induce senescence without cell death, while higher doses trigger apoptosis due to excessive stress [45]. The lack of senescence in this study suggests cytotoxicity at these concentrations, as confirmed by poly-caspase and viability assays.

### 3.3. Curcumin Treatment Altered Gene Expression in U87 MG Cells

Despite extensive research into curcumin’s role in glioblastoma, its impact on global gene expression remains underexplored. Given its pleiotropic effects in tumorigenesis, studying global expression could reveal novel genetic targets and enhance our understanding of its therapeutic potential. This study highlights the multifaceted role of curcumin in cancer therapy by modulating genes involved in growth, survival, and immune responses. The up-regulation of genes like C1orf116 and uncharacterized loci (LOC124902513, LOC107985667, LOC100506358, LOC112268263, and LOC105375676) suggests unexplored pathways in cancer regulation. METTL21C (10.29), a methyltransferase, enhances protein modifications related to cell cycle control and apoptosis, aligning with curcumin’s epigenetic modulation [46]. PNLDC1 (10.14) and AMPD1 (10.05-fold), involved in RNA processing and purine metabolism, may disrupt cancer cell homeostasis, impairing growth [47,48]. The up-regulation of TBR1 (9.66), a transcription factor involved in neuronal development, suggests that curcumin may influence differentiation pathways—which is highly relevant in glioblastoma, where differentiation is often impaired [49].

A key finding in this study was the up-regulation of RUNX3 (8.81), a runt-related transcription factor with tumor-suppressive roles in multiple cancers. RUNX3 is frequently down-regulated in gastric, breast, colorectal, renal, and hepatocellular cancers through point mutations, promoter hypermethylation, and cytoplasmic translocation [50,51]. It also resides in a genomic region often deleted in various tumors, resembling classic tumor suppressor genes [52].

RUNX3 up-regulation in curcumin-treated cells likely contributed to reduced viability, migration, and increased apoptosis, aligning with its known tumor-suppressive function in gliomas. Glioma patient studies indicate that RUNX3 is progressively inactivated through promoter hypermethylation, with reduced protein expression associated with disease progression [53]. Mei et al. reported lower RUNX3 expression in glioblastoma tissue compared with normal brain, with re-expression suppressing the migration of U251 and U87 MG cells via MMP-2 inhibition [54]. Additionally, RUNX3 overexpression induced Bim-caspase-dependent apoptosis and G0/G1 cell cycle arrest, and suppressed Wnt/β-catenin signaling, thereby improving survival in a xenograft model [55,56].

Protein kinase C gamma (PRKCG), a gene identified in this study as involved in several signaling pathways, was up-regulated by a fold change of 8.81 compared with control cells following curcumin treatment. Pathway analysis revealed that PRKCG could be involved in focal adhesion molecules, tyrosine kinase inhibition, and cancer-related pathways, as well as choline metabolism in cancer. PRKCG, is a member of the protein kinase C family of enzymes whose activation mediates various signaling pathways that regulate the balance between cell survival and cell death [57]. This particular iso-enzyme, while previously accepted to be expressed solely in the brain, was found to be aberrantly expressed in some colon and breast cancer cells, where a role for it as a putative tumor suppressor has been suggested [58].

Emerging evidence highlights the tumor-suppressive roles of protein kinase C (PKC) isoenzymes, with most PKC mutations in cancers being not just loss-of-function but also dominant negative [57]. Their frequent inactivation suggests that restoring PKC activity could be a viable cancer treatment strategy. Satow et al. reported the expression of PKCγ (PRKCG) in normal colonic epithelia, with its down-regulation linked to poor patient outcomes [59].

In gliomas, PRKCG expression and methylation patterns correlate with tumor progression, with glioblastomas exhibiting the lowest expression and highest methylation [60]. Though PRKCG’s role in glioblastoma remains unclear, its up-regulation following curcumin treatment in this study suggests a potential anti-tumorigenic effect, possibly influencing key dysregulated signaling pathways in cancer.

Curcumin has been shown to modulate Wnt signaling, a pathway frequently overactivated in glioblastoma and other cancers [61]. As key receptors for frizzled proteins, transmembrane frizzled receptors drive canonical and non-canonical signaling, influencing proliferation, angiogenesis, migration, and invasion [62]. High expression of FZD1, 2, 5, 6, 7, and 8 correlates with oncogenic mTOR signaling and poor prognosis in gliomas [63].

In this study, curcumin treatment down-regulated the expression of five frizzled receptors: FZD2 (2.95), FZD8 (3.18), FZD1 (2.62), FZD7 (1.93), and FZD5 (1.99). Among these, FZD2 is a known prognostic marker for glioma progression, with its expression linked to tumor grade [64]. Ran et al. found that FZD2 suppression reduced glioblastoma cell stemness, proliferation, migration, and invasion, coinciding with the inhibition of the Notch and NF-κB pathways [65].

High expression of FZD1 and FZD8 has been linked to poor overall survival in glioblastoma patients [66,67]. In low-grade glioma, FZD8 was identified as a key driver of tumor recurrence [67]. Interestingly, its repression via bivalent modification in glioma tumorigenesis suggests a potential tumor-suppressive role [68]. While its function in glioblastoma remains unclear, aberrant FZD8 expression has been implicated in gastric, prostate, lung, pancreatic, and renal cancers [69,70]. Additionally, FZD8 activation of Wnt signaling contributes to chemoresistance in triple-negative breast cancer [71]. Whether FZD down-regulation in curcumin-treated cells suppressed tumorigenesis warrants further investigation.

Curcumin treatment influenced TGF-β family protein expression in this study. Growth differentiation factor (GDF) proteins, part of the TGF-β family, regulate cell differentiation, proliferation, and apoptosis [72]. Loss of differentiation, a hallmark of cancer, fuels unchecked proliferation, often driven by cancer stem cells [73]. Inducing differentiation limits proliferation, shifting cells to a therapy-sensitive, less metastatic state [74]. While GDF proteins have both tumorigenic and tumor-suppressive roles, their exact functions in cancer remain unclear [72]. GDF2 suppresses breast and ovarian cancer metastasis [75], while GDF9 (up-regulated 3.75-fold by curcumin) exhibits tumor-suppressive effects in bladder cancer [76]. Conversely, elevated GDF15 levels in glioblastoma patients are correlated with a poor prognosis, immune suppression, and increased invasion [77,78]. Interestingly, Kadowaki et al. reported that GDF15 overexpression in glioblastoma cells with low basal levels induced apoptosis [79]. In this study, GDF15 mRNA increased 1.52-fold with curcumin treatment, highlighting the complex and context-dependent roles of GDF and other TGFβ superfamily members.

## 4. Materials and Methods

### 4.1. Curcumin

Curcumin (95% purity) [(E, E)-1,7-bis(4-hydroxy-3-methoxyphenyl)-1,6-heptadiene-3,5-dione] was obtained from Sigma-Aldrich (St. Louis, MO, USA) and stored as a 5 mM stock solution in ethanol at −20 °C, protected from light.

### 4.2. Cell Culture

The human glioblastoma cell line (U87 MG) was obtained from the American Type Culture Collection (ATCC, Manassas, VA, USA). Cells were cultured in T75 or T175 flasks (Greiner, Monroe, NC, USA) using high-glucose Dulbecco’s modified Eagle medium (DMEM) (ATCC) supplemented with 10% fetal bovine serum (Atlas Biologicals, Fort Collins, CO, USA) and 1× antibacterial/antimycotic solution (Gibco, Grand Island, NY, USA). Cultures were maintained in a humidified incubator at 37 °C with a gas mixture of 5% CO_2_ and 95% air.

### 4.3. Cell Viability Assay

The impact of increasing curcumin concentrations on the viability of U87 MG cells was evaluated. A total of 5 × 10^3^ cells were seeded per well in a 96-well microplate with 100 μL of media and incubated overnight. Cells were then treated with curcumin at concentrations of 10, 20, 30, 40, and 50 μmol/L, along with ethanol as the vehicle control (0.1%), for 24, 48, and 72 h. Cell viability was assessed using the Cell Counting Kit-8 (CCK-8; Dojindo Molecular Technologies, Inc., Kumamoto, Japan) assay. Briefly, after treatment, 10 μL of CCK-8 solution was added to each well containing 100 μL of treatment media, followed by a 4 h incubation. Optical density was then measured at 450 nm using the SpectraMax iD3 Multi-Mode Microplate Reader (Molecular Devices, San Jose, CA, USA). The cytotoxicity of curcumin was determined by calculating the IC50 from the dose-response curve obtained through the cell viability assay at 24 h.

### 4.4. Poly-Caspase Assay

To examine curcumin-induced apoptosis in U87 MG cells, caspase enzyme activity was measured using the poly-caspase assay (FAM FLICA, ImmunoChemistry Technologies, Davis, CA, USA). Cells were cultured in T25 flasks with phenol red-free Dulbecco’s modified Eagle’s medium (Gibco, Grand Island, NY, USA) supplemented with 10% FBS and 1× antibiotic/antimycotic solution. Once cells reached 80% confluence, they were treated with ethanol (vehicle control) or 10 or 20 μmol/L curcumin. Staurosporine (5 μmol/L) (ImmunoChemistry Technologies, Bloomington, MN, USA) served as a positive control. After 24 h of treatment, cells were collected, stained with FLICA, and incubated for one hour at 37 °C. Following washing, a 100 µL aliquot of the cell suspension, at a density of greater than 2 × 10⁶ cells/mL, was transferred to a 96-well flat-bottom microplate. Fluorescence was measured at an excitation wavelength of 488 nm and an emission wavelength of 520 nm using a microplate reader.

### 4.5. SA-Beta Galactosidase Assay

To determine whether curcumin treatment induced or inhibited senescence, β-galactosidase—a pH-dependent enzyme marker of senescent cells—activity was measured using the SA-β-galactosidase assay (Cell Biolabs, San Diego, CA, USA). Cells were cultured in 6-well plates with phenol red-free Dulbecco’s modified Eagle’s medium supplemented with 10% FBS and 1× antibiotic/antimycotic solution. Once cells reached 80% confluence, they were treated with ethanol (vehicle control) or 10 or 20 μmol/L curcumin. After 24 h of treatment, cells were collected, stained, and transferred to 96-well flat black-bottom microplates. Fluorescence (optical density) was measured at 360/465 nm (excitation/emission) using a microplate reader, and the results were expressed as relative fluorescence units (RFU).

### 4.6. RNA Isolation and Library Preparation

The effect of curcumin treatment on global gene expression in U87-MG cells was assessed using RNA-seq. Three replicates each of two experimental controls were set up: negative control (vehicle-treated cells) and treated cells. Cells were cultured in 60 mm × 15 mm cell culture dishes (Wuxi NEST Biotechnology Co., Ltd., Wuxi, Jiangsu, China) until 80–90% confluence and treated with 20 μmol/L curcumin. After incubation for 6 h, RNA was isolated using the RNeasy Plus Mini Kit (QIAGEN, Germantown, MD, USA) and stored at −20 °C until sequencing. Further library preparation followed protocol in [6].

### 4.7. RNA-Seq Analysis

Sequencing reads were processed by trimming adapters and removing low-quality bases (Phred score < 30) using Trimmomatic v0.39 [80]. The filtered reads were then aligned to the human reference genome (GRCh38.p13) using the STAR RNA-Seq aligner v2.7.11a [81], generating BAM alignment files. Read quantification was performed with the HTSeq R package (version 2.0.3) [82] using genome annotations in GFF format. Differential gene expression (DEG) analysis was conducted using DESeq2 [83], considering genes with a log2FoldChange ≥ 1 and a false discovery rate (FDR) of ≤0.05. Pathway enrichment analysis was carried out using KOBAS [84].

### 4.8. Statistical Analysis

All statistical analyses were conducted using GraphPad Prism v10.4.0.621 for Windows (GraphPad Software, Boston, MA, USA, www.graphpad.com, accessed on 1 December 2024). A *p*-value of <0.05 was considered statistically significant.

## 5. Conclusions

In conclusion, this study elucidated that curcumin acts as a promising therapeutic agent against U87 glioblastoma cells by targeting multiple cancer-related processes, including regulation of the cell cycle, suppression of cell viability, induction of pro-apoptotic genes, down-regulation of anti-apoptotic genes, and activation of anti-metastatic genes. Curcumin exerts these effects through modulation of several critical signaling pathways, such as the calcium signaling pathway; the PI3K-Akt signaling pathway; TNF signaling; MAPK, Ras, TGF-β, Wnt, and cytokine signaling, and regulation of the actin cytoskeleton. Furthermore, curcumin influences the expression of several pivotal genes involved in apoptosis, metastasis, and stem cell dynamics, including AIFM3, TP53AIP1, CRD14, NIBAN3, BOK, BCL2L10, BCL2L14, BNIPL, FASLG, GZMM, TNFSF10, TNFSF11, TNFSF4, FOS, PRKCG, GDF7, GDF9, GDF15, GDF5, FZD1, FZD2, FZD8, SMAD5, APC, WNT7B, and HOXA1. By modulating these interconnected molecular networks, curcumin impairs the survival of cancer stem cells, reduces therapy resistance, inhibits metastatic potential, and sensitizes tumor cells to conventional treatments. Collectively, these findings highlight curcumin’s multifaceted role in cancer prevention and therapy, emphasizing its potential as a powerful adjunct in glioblastoma management.

## Data Availability

The raw paired-end Illumina RNA-sequencing reads generated in the current study are available in the Sequence Read Archive (SRA) at NCBI under the Bio project accession numbers PRJNA1243709.

## Supplementary Materials

The following supporting information can be downloaded at: https://www.mdpi.com/article/10.3390/molecules30102108/s1, Figure S1: Cell viability was assessed at 24 h following treatment with various concentrations of curcumin, and the corresponding IC_50_ value was determined based on the dose–response relationship. Figure S2: Principal Component Analysis (PCA) of RNA-seq data. PCA shows the variance in normalized gene expression profiles across samples. The first two principal components, PC1 and PC2, explain 85% and 5% of the total variance, respectively. Samples are color- and shape-coded by group: U87C (control) in red circles and U87T (treated) in cyan triangles.
