# Supplementary material for: Curcumin-Induced Molecular Mechanisms in U-87 MG Glioblastoma Cells: Insights from Global Gene Expression Profiling
Source: Molecules. 2025 May 9;30(10):2108. doi: 10.3390/molecules30102108 (PMC12113757; doi:10.3390/molecules30102108)
Supplement: Supplementary file 1 [file molecules-30-02108-s001.zip › molecules-3597100-supplementary.pptx]

## Slide 1
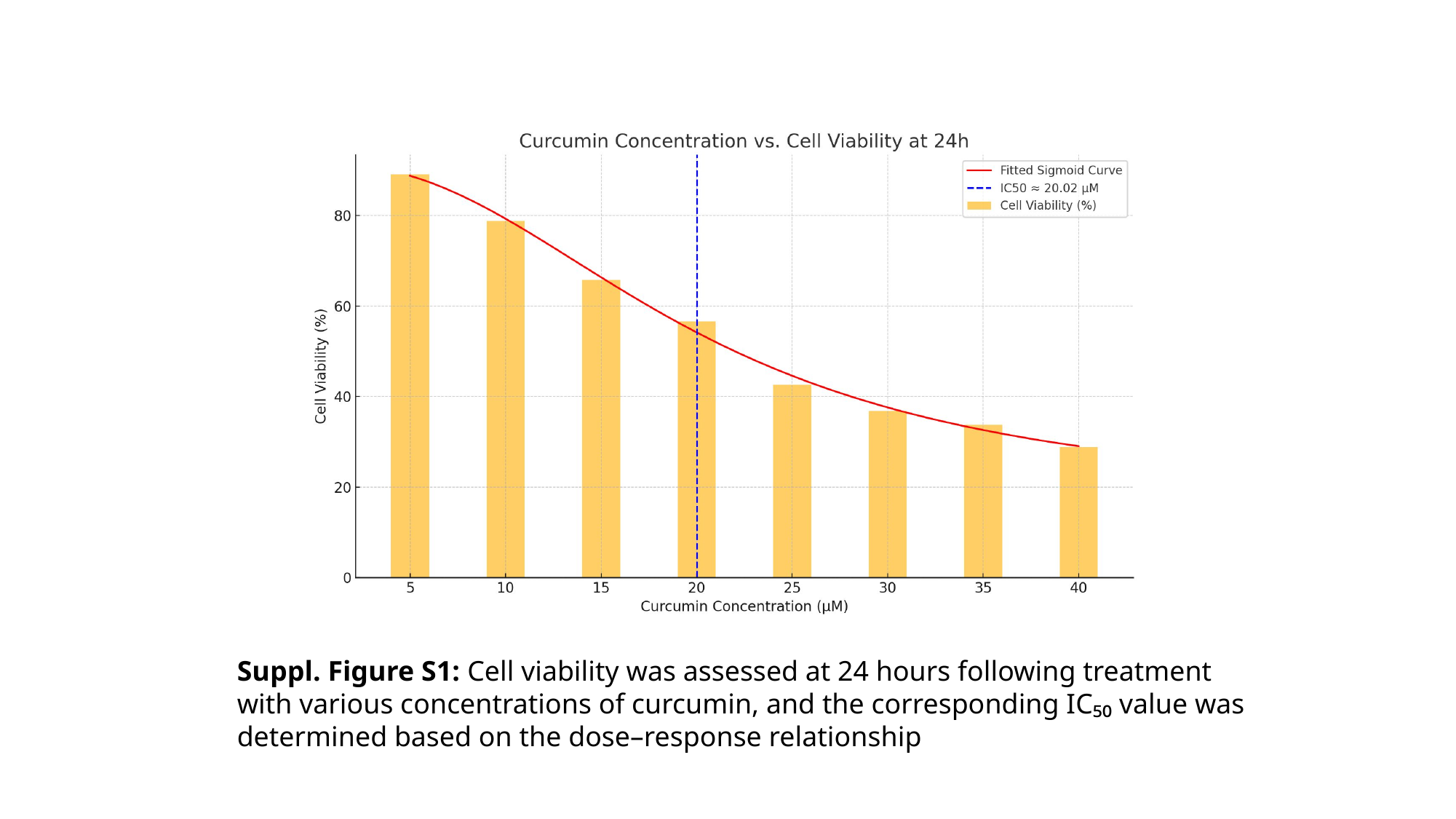

Suppl. Figure S1: Cell viability was assessed at 24 hours following treatment with various concentrations of curcumin, and the corresponding IC₅₀ value was determined based on the dose–response relationship

## Slide 2
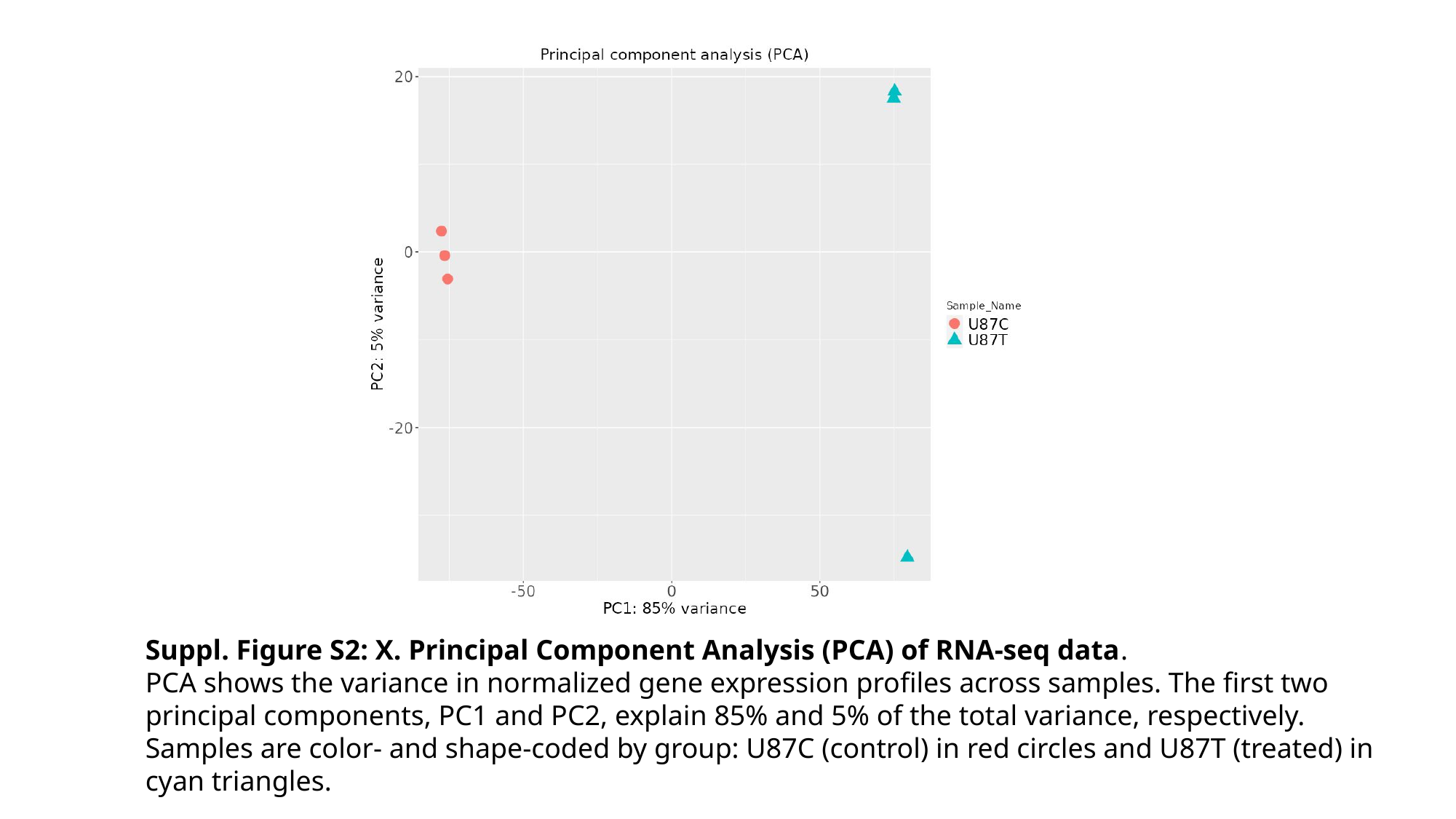

Suppl. Figure S2: X. Principal Component Analysis (PCA) of RNA-seq data.
PCA shows the variance in normalized gene expression profiles across samples. The first two principal components, PC1 and PC2, explain 85% and 5% of the total variance, respectively. Samples are color- and shape-coded by group: U87C (control) in red circles and U87T (treated) in cyan triangles.
